# Elevated Levels of CTRP1 in Obesity Contribute to Tumor Progression in a p53-Dependent Manner

**DOI:** 10.3390/cancers13143619

**Published:** 2021-07-19

**Authors:** Rackhyun Park, Minsu Jang, Yea-In Park, Yeonjeong Park, Sim Namkoong, Jin I. Lee, Junsoo Park

**Affiliations:** 1 Division of Biological Science and Technology, Yonsei University, Wonju 26493, Korea; rockhyun@yonsei.ac.kr (R.P.); minsujang@yonsei.ac.kr (M.J.); pyi012324@yonsei.ac.kr (Y.-I.P.); bbling408@yonsei.ac.kr (Y.P.); jinillee@yonsei.ac.kr (J.I.L.); 2 Department of Biochemistry, Kangwon National University, Chuncheon 24341, Korea; simn@kangwon.ac.kr

**Keywords:** CTRP1, obesity, p53, cancer, adiponectin

## Abstract

**Simple Summary:**

Obesity is regarded as a risk factor for various cancers. However, the molecular mechanisms linking obesity with cancer remain primarily uncharacterized. In this study, we demonstrate that CTRP1, an adiponectin paralogue, promotes tumor growth in a p53-dependent manner. Obese mice on a high-fat diet showed a higher level of CTRP1 protein in serum. It is also known that CTRP1 treatment contributes to tumor growth and cell migration. These results indicate that an elevated level of CTRP1 in obesity promotes tumor progression.

**Abstract:**

Mounting evidence supports the relationship between obesity and cancer. However, the molecular mechanisms linking obesity with cancer remain largely uninvestigated. In this study, we demonstrate that the expression of C1q/TNF-related protein 1 (CTRP1), an adiponectin paralogue, contributes to tumor growth by regulating the tumor suppressor p53. In our study, obese mice on a high-fat diet showed higher serum CTRP1 levels. Through in vitro experiments, we showed that the secreted form of CTRP1 in the culture medium decreased p53 expression and p53-dependent transcription in the cells. Moreover, CTRP1 treatment enhanced colony formation and cell migration. These results collectively suggest that elevated levels of CTRP1 in obesity significantly contribute to tumor progression.

## 1. Introduction

Obesity is regarded as a risk factor for various cancers, and cancer incidence increases in the prevalence of risk factors such as obesity [1,2,3]. Recent reports show how obesity is associated with an increased incidence of at least 13 different cancers, including the following: endometrial, esophageal, renal, and pancreatic adenocarcinomas, as well as hepatocellular carcinoma, gastric cardia cancer, meningioma, multiple myeloma, and colorectal, postmenopausal breast, ovarian, gallbladder, and thyroid cancers [4]. Some of the probable biological mechanisms linking obesity and cancer include (1) insulin resistance and abnormal IGF-1 signaling, (2) sex hormone signaling, (3) subclinical chronic low-grade inflammation, and (4) alterations in the levels of adipocyte-derived factors among others [5,6,7,8].

p53 is an important tumor suppressor gene that regulates apoptosis, cell cycle, and cellular senescence [9]. While p53 is mutated in up to 50% of human cancers, wild-type p53 is functionally inactivated in various cancers by several mechanisms, such as promoter methylation and ubiquitin-mediated degradation [10]. Growth promoting signals decrease the level of p53 to inactivate its tumor suppressor function [11]. MDM2, a typical p53 ubiquitin ligase, gets activated by Akt-mediated phosphorylation and leads to p53 degradation [12,13].

Adiponectin, secreted by white adipose tissues, belongs to the adipokine family and the adiponectin transcript is most abundant in adipose tissue [14,15,16]. Adiponectin has insulin-sensitizing, anti-inflammatory, antiatherogenic, proapoptotic, and antiproliferative effects [17]. The target cells activated by adiponectin through cognate receptors show activation of many cell signaling pathways, such as AMPK, mTOR, Akt, MAPK, STAT3, and NF-κB, contributing to the inhibition of carcinogenesis [17,18,19]. Complement 1q tumor necrosis factor-related protein 1 (CTRP1) belongs to a family of adiponectin paralogues and is mainly expressed in the stromal vascular fraction of adipose tissues [20]. Similar results are seen in obese mice that express higher plasma levels of CTRP1, which positively correlates with a higher body mass index (BMI) [21,22]. CTRP1 has been reported to decrease blood glucose levels in mice, with CTRP1 transgenic mice showing decreased high-fat diet-induced weight gain [23]. Structural studies have revealed that CTRP1 protein contains a globular C1q/TNF domain and collagen domain and can form a trimer [24]. This report highlights how the elevated serum level of CTRP1 protein in obese mice contributes to tumor progression by modulating the p53-dependent pathway.

## 2. Materials and Methods

### 2.1. Cell Culture, Cell Proliferation Assay, Clonogenic Assay, and Cell-Conditioned Media

HCT116 human colorectal cancer cells, MCF7 human breast cancer cells, A549 human lung cancer cells, H1299 human lung cancer cells, and HEK 293T human embryonic kidney cells were maintained in DMEM (Welgene, Seoul, Korea) containing 10% fetal bovine serum (Thermo Fisher Scientific, Waltham, MA, USA) and an antibiotic-antimycotic solution (Welgene). Cell proliferation was measured by the MTT [3-(4,5-dimethylthiazol-2-yl)-2,5-diphenyltetrazolium bromide] assay. HCT116, MCF7, and A549 cells were seeded in 24-well culture plates (2 × 10^5^ cells/well). After 24 h of plating, MTT solution was added at a final concentration of 1 mg/mL, and the mixture was incubated for 3 h. MTT was purchased from USB Corporation (Cleveland, OH, USA). A clonogenic assay was performed using crystal violet. HCT116, MCF7, and A549 cells were seeded in 6-well culture plates (10^3^ cells/well). After 10 days of plating, the cells were fixed with 100% methanol and incubated for 10 min at –20 °C. Cells were then stained with crystal violet solution (20% methanol and 0.25% crystal violet) for 30 min. Crystal violet was purchased from Sigma-Aldrich (St. Louis, MO, USA). Cell conditioned media was collected from control lentivirus- and CTRP1 lentivirus-infected A549 cells. These cells were incubated for 48 h in serum-free media before collecting the conditioned media. The secreted protein-containing supernatant media was collected and filtered through a 0.45 µm filter. After filtration, this media was added to the cells.

### 2.2. Virus Production and Transduction

Lentivirus was prepared by co-transfection of the lentiviral transfer vector with the psPAX2 envelope and pMD2.G packaging plasmids into HEK 293T cells using the calcium phosphate transfection method (2M CaCl_2_, 2X HEPES buffered saline (pH 7.2)) [25]. The media was changed 12 h after transfection. The virus-containing supernatant media was collected 48 h and 72 h after transfection and filtered through a 0.45 µm filter. After filtration, the media was concentrated using a Lenti-X concentrator (Clontech, Mountain View, CA, USA). Cells were infected in media containing 8 µg/mL polybrene (Sigma-Aldrich, St. Louis, MO, USA).

### 2.3. Western Blotting

Cells were harvested and resuspended in cell lysis buffer (150 mM NaCl, 50 mM HEPES (pH 7.5), 1% NP40) containing a protease inhibitor cocktail (Roche, Basel, Switzerland). Whole-cell lysates were resolved by SDS-PAGE and transferred to PVDF membranes (GE Healthcare, Uppsala, Sweden) via Western blotting. Proteins were detected with a 1:1000 or 1:5000 dilution of the primary antibody using a chemiluminescence system (Dogen, Seoul, Korea). Images were acquired using the LAS4000 system (GE Healthcare, Uppsala, Sweden). The CTRP1 antibody was purchased from Invitrogen (Carlsbad, CA, USA), p21 antibody from Cell Signaling Technology (Danvers, MA, USA), and p53 antibody from Santa Cruz Biotechnology (Dallas, TX, USA). FR180204, ERK inhibitor, was purchased from Calbiochem (San Diego, CA, USA).

### 2.4. Immunofluorescence and Confocal Microscopy

Cells were grown on sterilized glass coverslips. The cells were then fixed with 4% paraformaldehyde. For immunostaining, cells were blocked with 3% BSA in PBS, stained with a 1:1000 dilution of the primary antibody in PBS, and stained with 1:1000 Alexa 488-conjugated secondary antibody (Invitrogen, Carlsbad, CA, USA) or Alexa 568-conjugated secondary antibody (Invitrogen, Carlsbad, CA, USA). Images were captured using a Carl Zeiss LSM710 confocal microscope (Carl Zeiss, ObERKochen, Germany). The Image J software was used to analyze cell images (https://imagej.nih.gov/ij, accessed on 2 June 2021).

### 2.5. Quantitative RT-PCR

Cells were harvested, and RNA was extracted using Trizol (Invitrogen, Carlsbad, CA, USA) following the manufacturer’s instructions. Equal amounts of RNA were then subjected to reverse transcription-PCR (RT-PCR) using the StepOnePlus Real-Time PCR System (Applied Biosystems, Foster City, CA, USA). The primer sequences were used for the following genes: p53-forward primer 5- CCC AAG CAA TGG ATG ATT TGA -3 and reverse primer 5- GGC ATT CTG GGA GCT TCA TCT -3′. p21-forward primer 5- CAT GTG GAC CTG TCA CTG TCT TGT A -3 and reverse primer 5- GAA GAT CAG CCG GCG TTT G -3. GADD45α-forward primer 5- GAG AGC AGA AGA CCG AAA GGA -3 and reverse primer 5- CAG TGA TCG TGC GCT GAC T -3, PIG3-forward primer 5- AAG GAA ATA ACC ACC ATG TTA GCC GTG CAC -3 and reverse primer 5- CTG GGG CAG TTC CAG GAC GAT CTT -3′, NOXA-forward primer 5- ACC AAG CCG GAT TTG CGA TT -3 and reverse primer 5- ACT TGC ACT TGT TCC TCG TGG, SESTRIN2-forward primer 5- AGA GGG CAC AGG AAA GAA -3 and reverse primer 5- TCA AGC ATA AAG GAC CAA A -3. The input mRNA was normalized via the amplification of RPL4 mRNA with the forward primer 5- GCT CTG GCC AGG GTG CTT TTG -3 and reverse primer 5- ATG GCG TAT CGT TTT TGG GTT GT -3′.

### 2.6. Animal and High-Fat Diet-Induced Obesity Mouse Model

Male C57BL/6 mice (6-week-old) were obtained from DBL (Seoul, Korea) and fed a lean control diet (Envigo, Indianapolis, IN, USA) or a high-fat diet (Research Diets, New Brunswick, NJ, USA) for 10 weeks. After 10 weeks, the control and high-fat diet mice were sacrificed, and blood was collected from the mice after anesthesia by puncturing the heart. Blood serum was isolated via centrifugation at 1500× *g* for 15 min following 30 min of clotting at room temperature. 

### 2.7. Statistical Analysis

Statistical significance of differences, during analysis of Western blotting, quantitative RT-PCR, and MTT assay results, was evaluated via a two-tailed *t*-test using Excel software (Microsoft, Seattle, WA, USA). In most experiments, statistical significance was set at *p* < 0.05.

## 3. Results

### 3.1. The Level of CTRP1 in Serum Is Elevated in Obesity

**Figure 1:**
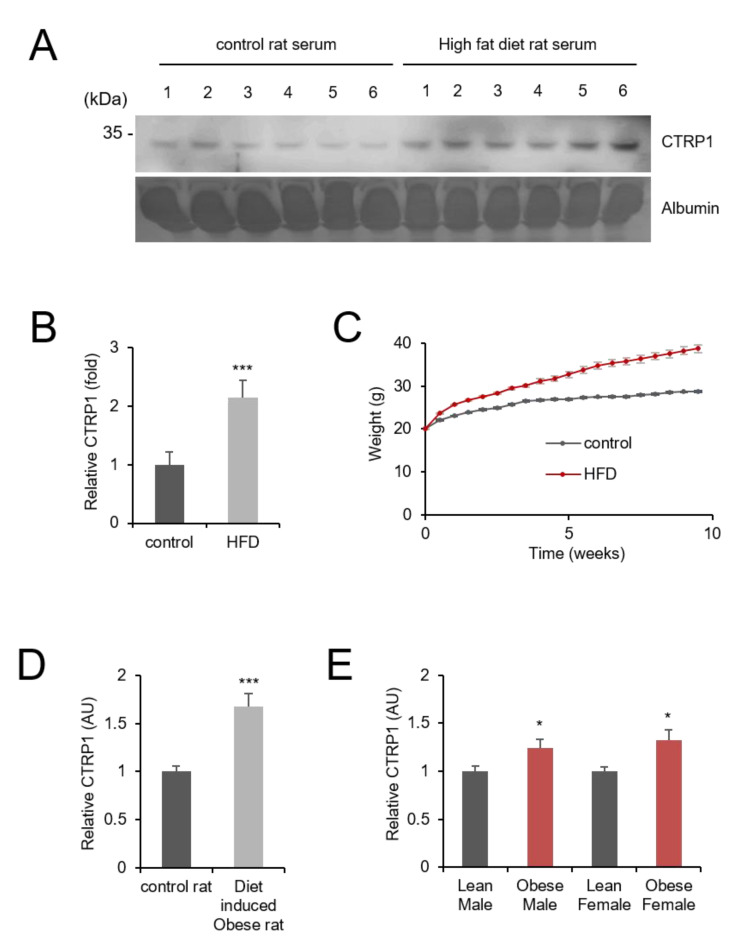
CTRP1 serum level is elevated in obesity. (**A**) The protein level of CTRP1 in serum is increased in high-fat diet mice. Mice were fed with a control diet or a high-fat diet for 10 weeks. After feeding, an equal amount of mouse blood serum was subject to Western blotting with anti-CTRP1 antibody (upper panel). Albumin was visualized by Ponceau S staining (bottom panel). Images of the uncropped western blots can be found in Figure S1. (**B**) The level of CTRP1 protein was quantified and depicted in the graph. The graph shows the average and standard error. Control vs high-fat diet rat, *** *p* < 0.005 (*n* = 6). (**C**) The weights of the mice are shown in the graph. (**D**) GEO data analysis shows an elevated level of CTRP1 mRNA in obese rats. GEO data were used for analysis. (**E**) GEO data shows that the level of CTRP1 mRNA is elevated in obese humans (abdominal subcutaneous adipocytes), * *p* < 0.05.

We used a high-fat diet-induced obese mouse model to identify the relationship between serum CTRP1 levels and obesity. The high-fat diet resulted in higher weight gain in mice when compared with the ones fed with a normal diet (Figure 1C). We analyzed serum CTRP1 expression in mice using Western blotting, which showed significantly higher CTRP1 levels in the serum of the obese mice (Figure 1A,B). To assess CTRP1 expression in obese animals, we used the GEO profiles database in NCBI (https://www.ncbi.nlm.nih.gov/geoprofiles/, accessed on 2 June 2021). In rats, CTRP1 mRNA levels were elevated in diet-induced obese mice epididymal fat (Figure 1D) [26]. In humans, CTRP1 mRNA levels were found to be higher in abdominal adipocytes of obese people than in abdominal adipocytes of normal people, irrespective of their sex (Figure 1E) [27].

### 3.2. Increased CTRP1 Expression Downregulates the Level of p53 Protein and p53-Dependent Transcription

**Figure 2:**
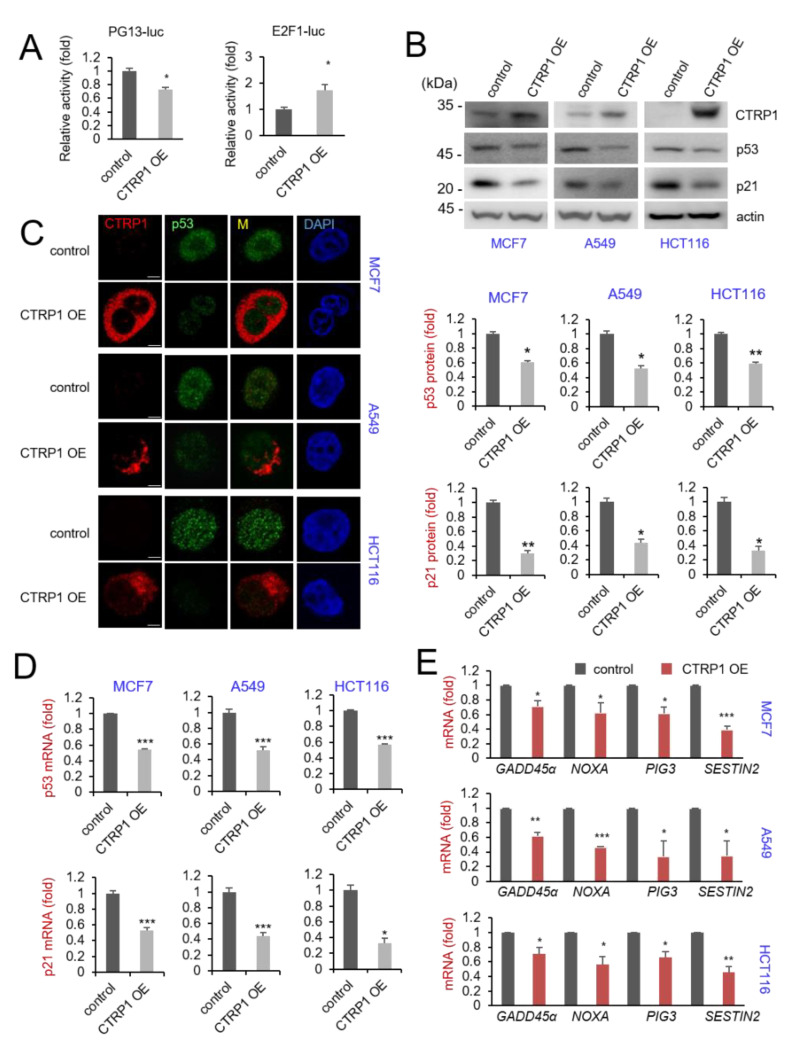
CTRP1 overexpression (OE) decreases p53 level and p53-dependent transcription. (**A**) Overexpression of CTRP1 decreased p53-dependent luciferase reporter activity in MCF7 cells. MCF7 cells were transfected with p53-dependent reporter (PG13-Luc) or E2F1-dependent reporter (E2F1-Luc) with either a control plasmid or the CTRP1 overexpression plasmid. Control vs CTRP1 overexpression, * *p* < 0.05. (**B**) Lentivirus-mediated CTRP1 overexpression decreased p53 expression. MCF7 cells, A549 cells, and HCT116(+/+) cells were infected with either control lentivirus or lentivirus encoding CTRP1. At 72 h after the infection, cells were selected by puromycin, harvested, and equal amounts of proteins from the cell lysates were probed with the indicated antibodies (upper panel). The level of p53 and p21 protein was quantified and depicted in the graph (lower panel). Control vs CTRP1 overexpression, * *p* < 0.05, ** *p* < 0.01. Images of the uncropped western blots can be found in Figure S2. (**C**) CTRP1 decreases p53. MCF7 cells, A549 cells, and HCT116(+/+) cells were infected with either control lentivirus or lentivirus encoding CTRP1, and the cells were fixed and immunostained with anti-p53 antibody (green) and anti-CTRP1 antibody (red). Scale bar, 10 μm. (**D**) CTRP1 overexpression decreased the mRNA level of p53 and p21. The level of p53 and p21 mRNA was examined by quantitative RT-PCR in control and CTRP1 overexpressed cells. * *p* < 0.05, *** *p* < 0.005. (**E**) The expression levels of GADD45α, NOXA, PIG3, and SESN2 mRNAs were measured using quantitative RT-PCR * *p* < 0.05, ** *p* < 0.01, *** *p* < 0.005.

Next, we used luciferase reporter constructs containing various promoters of signaling molecules to examine the cellular function of CTRP1. MCF7 cells were transiently co-transfected with CTRP1 overexpression plasmid (CTRP1OE) with the various reporter constructs. The assays showed that CTRP1 overexpression decreased PG13-luciferase activity (p53-dependent signaling) and increased E2F1-dependent luciferase activity (Figure 2A).

Next, we examined whether CTRP1 expression decreased p53 protein levels and p53-dependent transcription. Western blot analysis showed that the expression levels of p53 protein and p21 protein, a well-known transcriptional target of p53, were significantly reduced in CTRP1 overexpressed cancer cell lines MCF7, A549, and HCT116 (Figure 2B). Confocal microscopy confirmed that the overexpression of CTRP1 downregulated p53 protein expression (Figure 2C). Our investigation also revealed that CTRP1 overexpression inhibited the expression of p53 and p21 at the transcript level. (Figure 2D). We also examined the mRNA expression levels of p53-dependent genes NOXA, GADD45α, PIG3, and SESTRIN2, which exhibited significant downregulation (Figure 2E). These results indicated that CTRP1 overexpression resulted in the decreased expression of p53 and p53-dependent genes.

**Figure 3:**
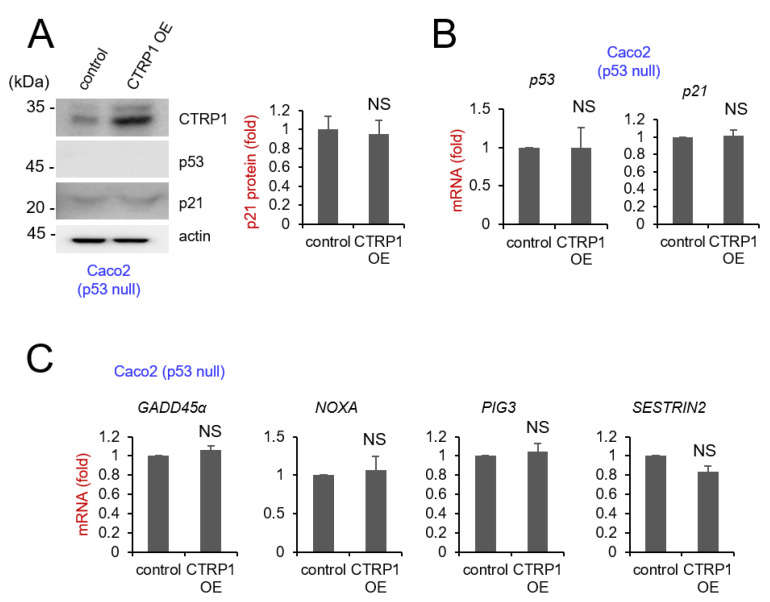
CTRP1 overexpression in Caco2, a p53 null cell line did not decrease p53 and p53-dependent transcription. (**A**) CTRP1 OE Caco2 cells did not show a decrease in p21 protein level. Caco2 cells were infected with either control lentivirus or CTRP1 encoding lentivirus. Seventy-two h after CTRP1 lentivirus infection, cells were harvested, and equal amounts of cell lysates were probed with the indicated antibodies (left panel), and the level of p21 protein was quantified and depicted in the graph (right panel). Images of the uncropped western blots can be found in Figure S3. (**B**) CTRP1 OE Caco2 cells did not decrease p53 and p21 mRNA level. The expression levels of p53 and p21 mRNAs were measured using quantitative RT-PCR. Control vs. CTRP1 OE in Caco2 cells. NS: Not Significant. (**C**) CTRP1 OE Caco2 cells did not decrease the mRNA level of p53-dependent transcription. The expression levels of GADD45α, NOXA, PIG3, and SESN2 mRNAs were measured using quantitative RT-PCR. NS: Not Significant.

We further tested this hypothesis by overexpressing CTRP1 in Caco2 cells, a p53 null cell line. Western blotting showed that the p53-dependent transcription of p21 did not decrease in this case (Figure 3A). Next, we examined the mRNA expression levels of p53 and p53 target genes by quantitative RT-PCR, and the levels of p53, p21, NOXA, GADD45, PIG3, and SESTRIN2 did not show any significant decrease (Figure 3B,C). These results indicated that CTRP1 decreased transcription of p53-dependent genes in the presence of p53.

### 3.3. Conditioned Media-Derived CTRP1 Decreased p53 and p53-Dependent Transcription

**Figure 4:**
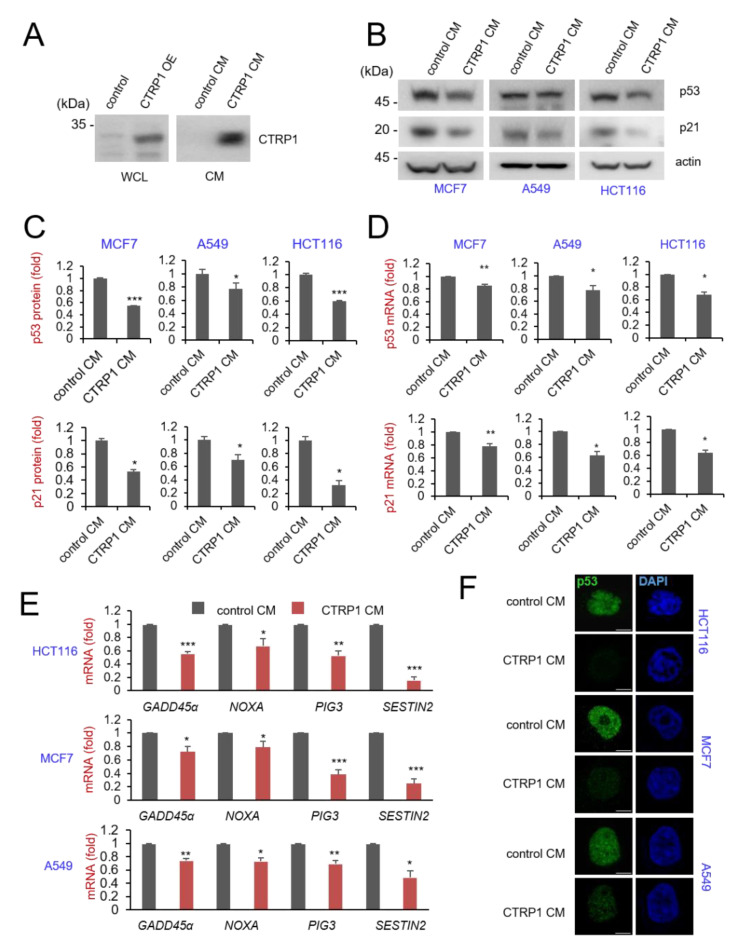
CTRP1 conditioned media decreased p53 and p53-dependent transcription. (**A**) CTRP1 conditioned media (CTRP1 CM) derived from CTRP1 overexpressed A549 cell lines. Lentivirus-mediated CTRP1 overexpression expresses CTRP1 protein in cell lysates (left panel). Cells were seeded and changed with CTRP1 overexpressed A549 media supernatant. At 48 h after changed, cells were harvested and probed with CTRP1 antibody (right panel). Images of the uncropped western blots can be found in Figure S4. (**B**) CTRP1 CM decreases p53 and p21 protein levels. HCT116 cells, MCF cells, and A549 cells were incubated with either control CM or CTRP1 CM. At 48 h after the incubation, cells were harvested and probed with the indicated antibodies. Images of the uncropped western blots can be found in Figure S5. (**C**) The level of p53 and p21 protein in each cell lines was quantified and depicted in the graph (* *p* < 0.05, ** *p* < 0.01, *** *p* < 0.005, below panel). (**D**) The level of p53 and p21 mRNA were quantified and depicted in the graph (* *p* < 0.05, ** *p* < 0.01, *** *p* < 0.005, below panel). (**E**) CTRP1 CM decreases p53 and p53-dependent transcripts. The expression level of *p53, p21, GADD45α, NOXA, PIG3*, and *SESN2* mRNAs was measured using quantitative RT-PCR in HCT116 cells, MCF7 A549 cells. (**F**) CTRP1 CM decreases p53 protein expression level. The HCT116 cells, MCF cells, and A549 were fixed and immunostained with anti-p53 (green). Scale bar, 10 μm.

Next, we demonstrated that the expression of CTRP1 affects the expression of p53 and p53-dependent transcription (Figure 2). Since CTRP1 is a secreted glycoprotein, we examined whether the secreted form of CTRP1 affects p53 and p53-dependent transcription. To determine the function of secreted CTRP1 protein, we collected conditioned media from A549 cells expressing CTRP1 protein and incubated MCF7, A549, and HCT116 cells with the conditioned media (Figure 4A). Incubation with the conditioned media resulted in the downregulation of p53 and p21 protein levels in MCF7, A549, and HCT116 cells, indicating that the secreted form of CTRP1 decreased p53 function (Figure 4B,C). A similar decrease in the mRNA levels of p53 and p21 was seen in the cells incubated with conditioned media (Figure 4D). We also found that the transcription of p53-dependent genes was downregulated by treating the cells with conditioned media-derived CTRP1 (Figure 4E). Finally, we used confocal microscopy to confirm the downregulation of p53 by conditioned medium-derived CTRP1 (Figure 4F). These results collectively indicate that secreted CTRP1 affects p53 expression and p53-dependent transcription.

### 3.4. CTRP1 Enhances Cell Proliferation and Migration

**Figure 5:**
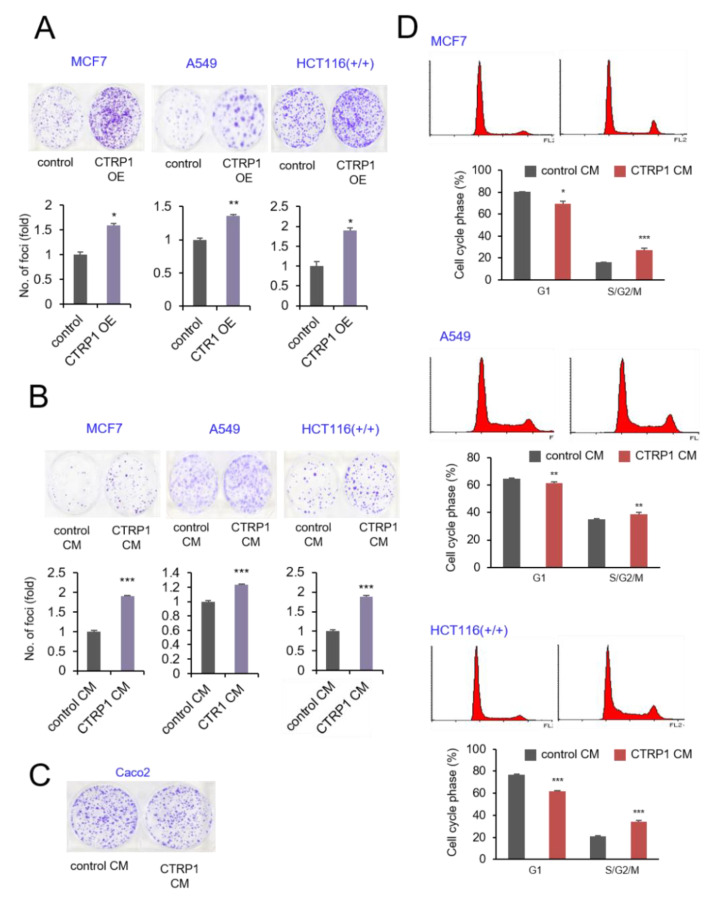
CTRP1 increases cell proliferation and migration of cancer cells. (**A**) CTRP1 overexpression increases cell proliferation. HCT116, MCF7, and A549 cells were infected with either control lentivirus or CTRP1 lentivirus. After puromycin selection, 1,000 cells were seeded and incubated for 10 days. After incubation, cell proliferation was measured by crystal violet assay. * *p* < 0.05, ** *p* < 0.01. (**B**) CTRP1 CM increase cell proliferation. HCT116, MCF7, and A549 cells were seeded at a density of 10^3^ cells per well and incubated for 10 days with either control CM or CTRP1 CM. After 10 days of incubation, cells were measured by crystal violet assay. *** *p* < 0.005. (**C**) CTRP1 CM did not increase the cell proliferation in p53 null cells (Caco2). (**D**) CTRP1 CM enhances cell cycle progression. Control CM and CTRP1 CM were analyzed by flow cytometry, and the cell cycle population (%) were calculated and shown in the graph * *p* < 0.05, ** *p* < 0.01, *** *p* < 0.005.

The crucial tumor-suppressive role of p53 led us to hypothesize that CTRP1 overexpression might result in increased tumor progression by inhibiting p53. We tested this hypothesis using a clonogenic assay. Lentivirus-mediated CTRP1 overexpression increased colony formation in MCF7, A549, and HCT116 cells (Figure 5A). Next, we incubated the cancer cells with conditioned media containing CTRP1 and found that the secreted form of CTRP1 also significantly increased colony formation (Figure 5B). However, incubation of the p53-null Caco2 cells with conditioned medium did not increase colony formation, indicating that CTRP1 increases colony formation in a p53-dependent manner (Figure 5C). These results indicated that CTRP1 positively regulates cell proliferation, thereby having a potential tumorigenic role. Since p53 also regulates the cell cycle, we examined whether CTRP1 contributes to cell cycle progression by regulating p53. When the cancer cells were incubated with CTRP1 conditioned medium, the percentage of S and G2/M phase cells was higher in MCF-7, A549, and HCT116 cells (Figure 5D). These data indicate that CTRP1 conditioned medium activates cell proliferation and cell cycle progression.

**Figure 6:**
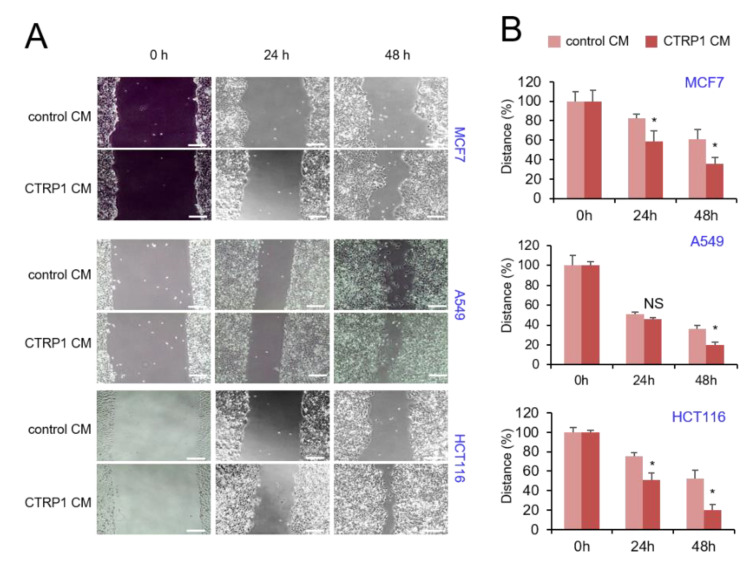
CTRP1 CM increases cell migration of cancer cells. (**A**) Cell migration of HCT116 cells, MCF7 cells, and A549 cells were measured with control CM or CTRP1 CM. Equal numbers of cells were plated in each well of a 6-well plate, and cells were scraped with a pipette tip. After the indicated times, images of the wound gaps were captured with a microscope. (**B**) The distance of the wound was analyzed by the Image J software. * *p* < 0.05.

Because p53 is an essential regulator of cell migration, we next examined the effect of CTRP1 on this cellular phenotype. The wound-healing assay showed that cell migration with CTRP1 conditioned medium was significantly faster than that of the control (Figure 6A,B). These results indicated that CTRP1 is also involved in cell migration.

### 3.5. CTRP1 Induces Cell Proliferation through the Activation of the ERK Signaling Pathway

**Figure 7:**
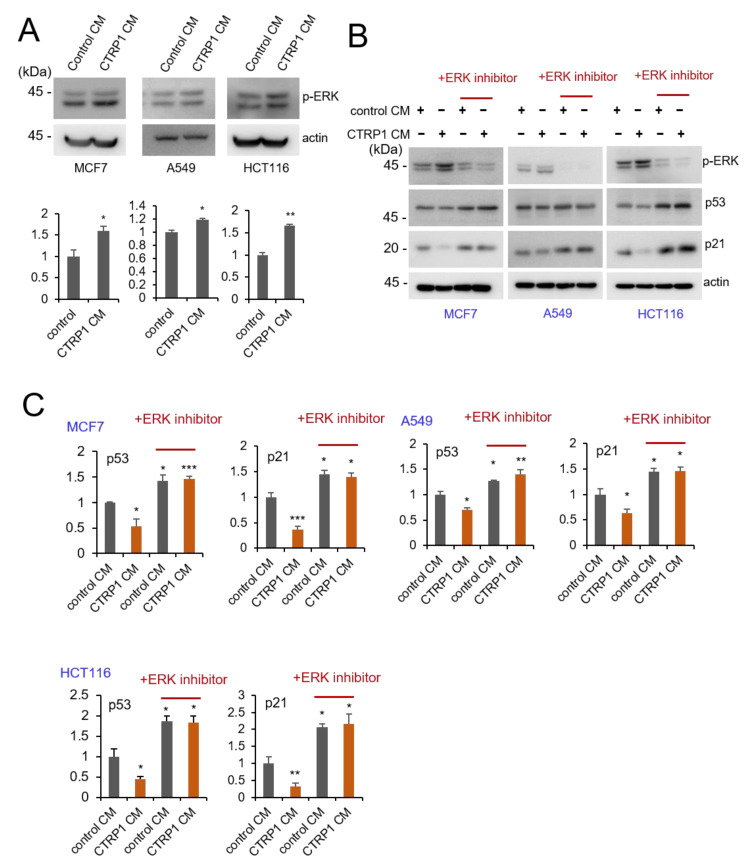
CTRP1 induces cell proliferation by activating the ERK signaling pathway. (**A**) CTRP1 CM increases p-ERK levels by activating the ERK signaling pathway. Control CM- and CTRP1 CM- treated HCT116 cells, MCF cells, and A549 cells were incubated with either control or ERK inhibitor. The level of p-ERK protein was quantified with an anti-p-ERK antibody and depicted in the graph (* *p* < 0.05, ** *p* < 0.01). Images of the uncropped western blots can be found in Figure S6. (**B**) ERK inhibitor treatment rescues p53 and p21 levels via inhibition of the ERK pathway. Cells were treated with either control or ERK inhibitor (FR180204, 10 μM) for 24 h. After treatment, cells were harvested and probed with the indicated antibodies. Images of the uncropped western blots can be found in Figure S7. (**C**) The level of p53 and p21 protein was quantified and depicted in the graph (* *p* < 0.05, ** *p* < 0.01, *** *p* < 0.005).

Finally, we examined the expression of various cell signaling proteins to determine the mechanism of regulation of p53 and p53-dependent transcription by CTRP1. Our investigations revealed that CTRP1 conditioned medium increased the levels of phospho-ERK protein in MCF7, A549, and HCT116 cells (Figure 7A). Since the activation of ERK signaling contributes to the downregulation of p53, we examined whether the inhibition of ERK signaling prevents p53 downregulation. We used FR180204 to inhibit Erk signaling and FR180204 treatment did not induce dramatic cell death at 10 µM. We found that ERK inhibitor treatment blocks CTRP1-mediated decrease in p53 and p21 protein (Figure 7B) and mRNA expression (Figure 7C). These results collectively indicate that ERK signaling is essential for CTRP1-mediated transcription of p53 and p53 target genes.

## 4. Discussion

In this study, we highlight the oncogenic role of CTRP1, a member of the adipokine paralogue family, and demonstrate how it activates cell proliferation by inhibiting p53 and p53-dependent transcription. CTRP1 was initially identified to be produced in adipose tissues, and we showed that the level of CTRP1 in mouse serum is elevated in high-fat diet-fed obese mice than in mice fed with a normal diet. Incubation of cancer cells with the secreted form of CTRP1 downregulated p53 and p53-dependent transcription. We also demonstrated that the secreted form of CTRP1 activates colony formation and cell cycle progression. Since the serum level of CTRP1 protein is elevated in obese mice, these results suggest that obesity-induced CTRP1 expression contributes to cancer progression.

Many reports support the hypothesis that obesity is positively correlated to tumor progression [1]. Adipose tissue secretes various adipokines, and their association with cancer is diverse. While adiponectin is inversely correlated to the progression of breast, colorectal, and endometrial cancers, leptin is associated with an increased risk of endometrial and renal cancers [28]. Here, we demonstrate that CTRP1, a protein secreted from adipose tissue, can activate tumor cell growth. While CTRP1 belongs to the adiponectin family, our data shows that the function of CTRP1 is different from that of the other adiponectins. In this study, we demonstrate that CTRP1 has the potential to activate tumor cell growth and migration.

p53 is the most important tumor suppressor, and we demonstrate that CTRP1 decreases p53 expression and p53-dependent transcription. We show how CTRP1 downregulates p53 in MCF-7, A549, and HCT116 cells, which express wild-type p53. However, such oncogenic effects of CTRP1 are not seen in a p53-null cell line (Figure 3 and Figure 5). These results indicate that the tumor-promoting effect of CTRP1 is wild-type p53-dependent. p53 mRNA expression was downregulated by CTRP1 overexpression and by the secreted form of CTRP1. The level of p53 protein is usually regulated by ubiquitin-mediated degradation. However, CTRP1 treatment downregulates p53 mRNA and protein levels (Figure 2 and Figure 4). These results suggest that CTRP1 regulates p53 at the transcriptional level, while treatment with an ERK inhibitor treatment increased p53 mRNA expression by abrogating the effects of CTRP1 (Figure 7). These results collectively support that ERK signaling downregulates p53 expression at the transcriptional level. Our novel finding suggests that secreted CTRP1 protein regulates p53 transcription by modulating the ERK signaling pathway. Further mechanistic study will be required to elucidate the function of CTRP1 protein.

Even though CTRP1 expression or secreted CTRP1 activates tumor cell proliferation in a p53-dependent manner, the role of CTRP1 extends far beyond the modulation of tumor progression by cancer cells. CTRP1 can act as a growth factor to promote cancer cell growth, similar to other growth factors. It is worth examining whether CTRP1 can induce tumor initiation, although our hypothesis suggests that it does not. Further research with CTRP1 knockout or transgenic mice will elucidate the long-term effects of CTRP1 on cancer initiation.

The findings from our study suggest that since elevated levels of CTRP1 contribute to tumor cell growth and migration, this adipocyte-secreted protein might be a potential link between obesity and carcinogenesis. Uncovering the molecular mechanisms of CTRP1 may provide a greater understanding of how obesity increases the risk of carcinogenesis. Further in-depth clinical studies are required to prove the correlation between serum CTRP1 levels and the degree of cancer progression.

## 5. Conclusions

Here, we demonstrate that obese mice on a high-fat diet showed higher serum CTRP1 levels, and the secreted form of CTRP1 in culture medium decreased p53 expression and p53-dependent transcription in cells. CTRP1 treatment enhanced colony formation and cell migration. These results indicate that elevated levels of CTRP1 in obesity contribute to tumor progression.

## Data Availability

Publicly available datasets were analyzed in this study. This data can be found here: https://www.ncbi.nlm.nih.gov/geoprofiles/GDS2946/1372590_at,GDS1496/48656_at, accessed on 2 June 2021.

## Supplementary Materials

The following are available online at https://www.mdpi.com/article/10.3390/cancers13143619/s1, Figure S1: Uncropped western blots from Figure 1A, Figure S2: Uncropped western blots from Figure 2B, Figure S3: Uncropped western blots from Figure 3A, Figure S4: Uncropped western blots from Figure 4A, Figure S5: Uncropped western blots from Figure 4B, Figure S6: Uncropped western blots from Figure 7A, Figure S7: Uncropped western blots from Figure 7B.
