# Supplementary material for: Elevated Levels of CTRP1 in Obesity Contribute to Tumor Progression in a p53-Dependent Manner
Source: Cancers (Basel). 2021 Jul 19;13(14):3619. doi: 10.3390/cancers13143619 (PMC8306638; doi:10.3390/cancers13143619)
Supplement: Supplementary file 1 [file cancers-13-03619-s001.zip › cancers-1280917-supplementary.pdf]

cancers

MDPI

# Supplementary Materials: Elevated Levels of CTRP1 in Obesity Contribute to Tumor Progression in A p53-Dependent Manner

Rackhyun Park, Minsu Jang, Yea-In Park, Yeonjeong Park, Sim Namkoong, Jin I. Lee and Junsoo Park

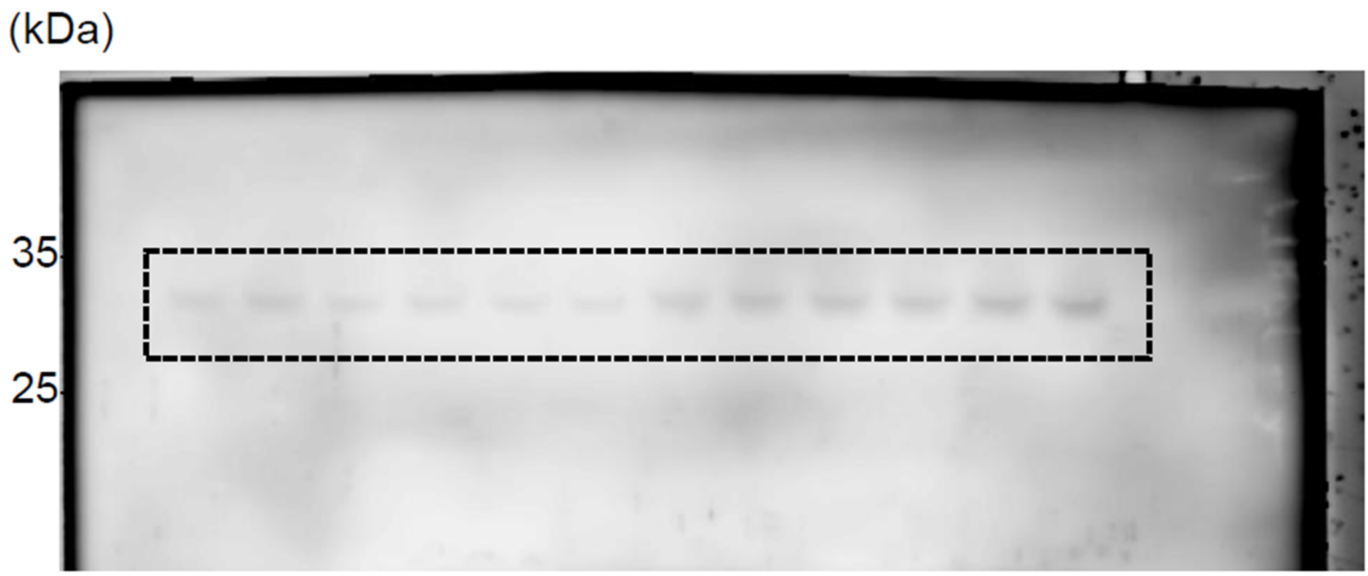


**Figure S1.** Uncropped western blots from Figure 1A. Uncropped western blot membranes, dotted lines indicate the cropped area used in Figure 1A western blot.

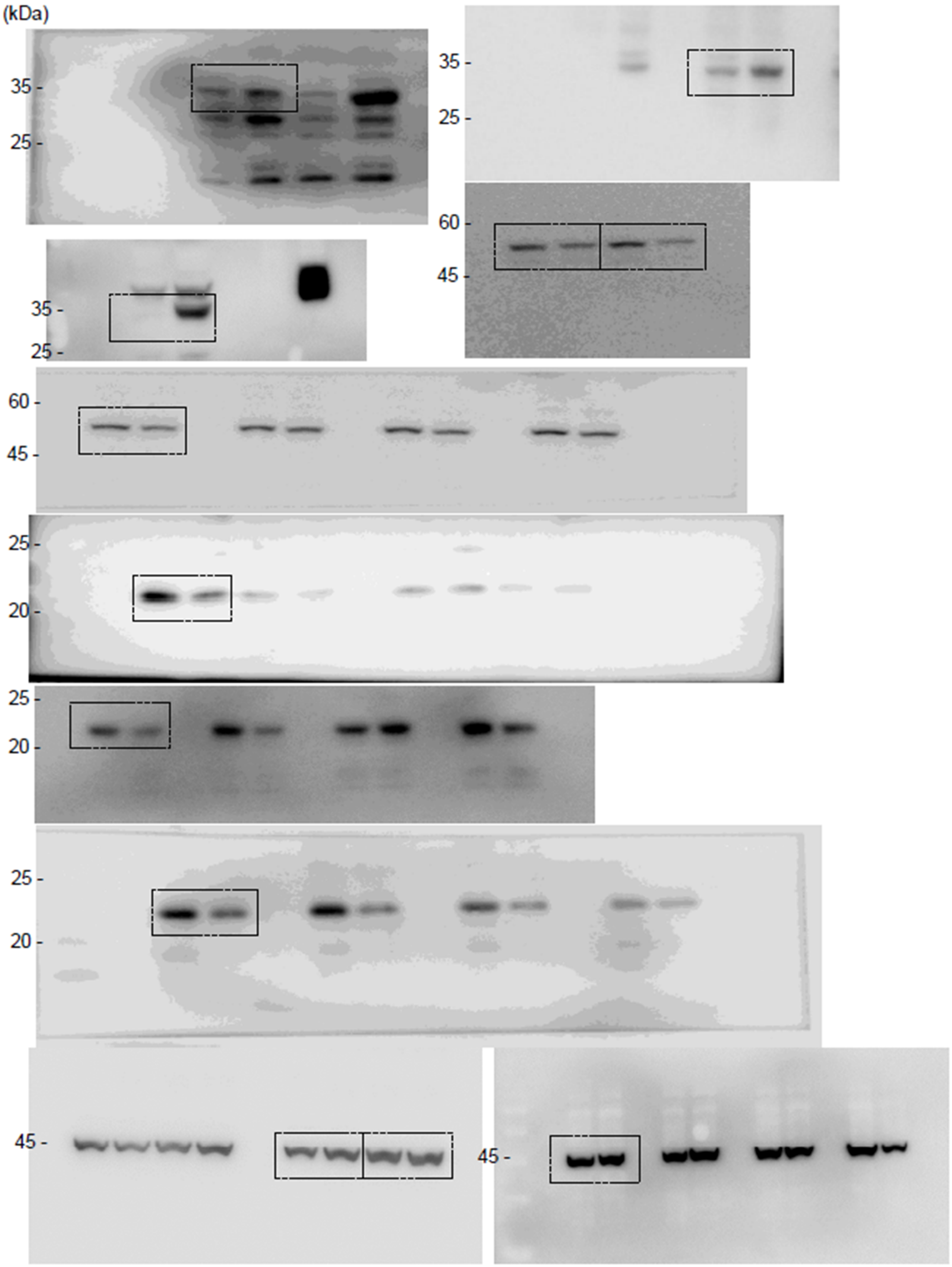


**Figure S2.** Uncropped western blots from Figure 2B. Uncropped western blot membranes, dotted lines indicate the cropped area used in Figure 2B western blot.

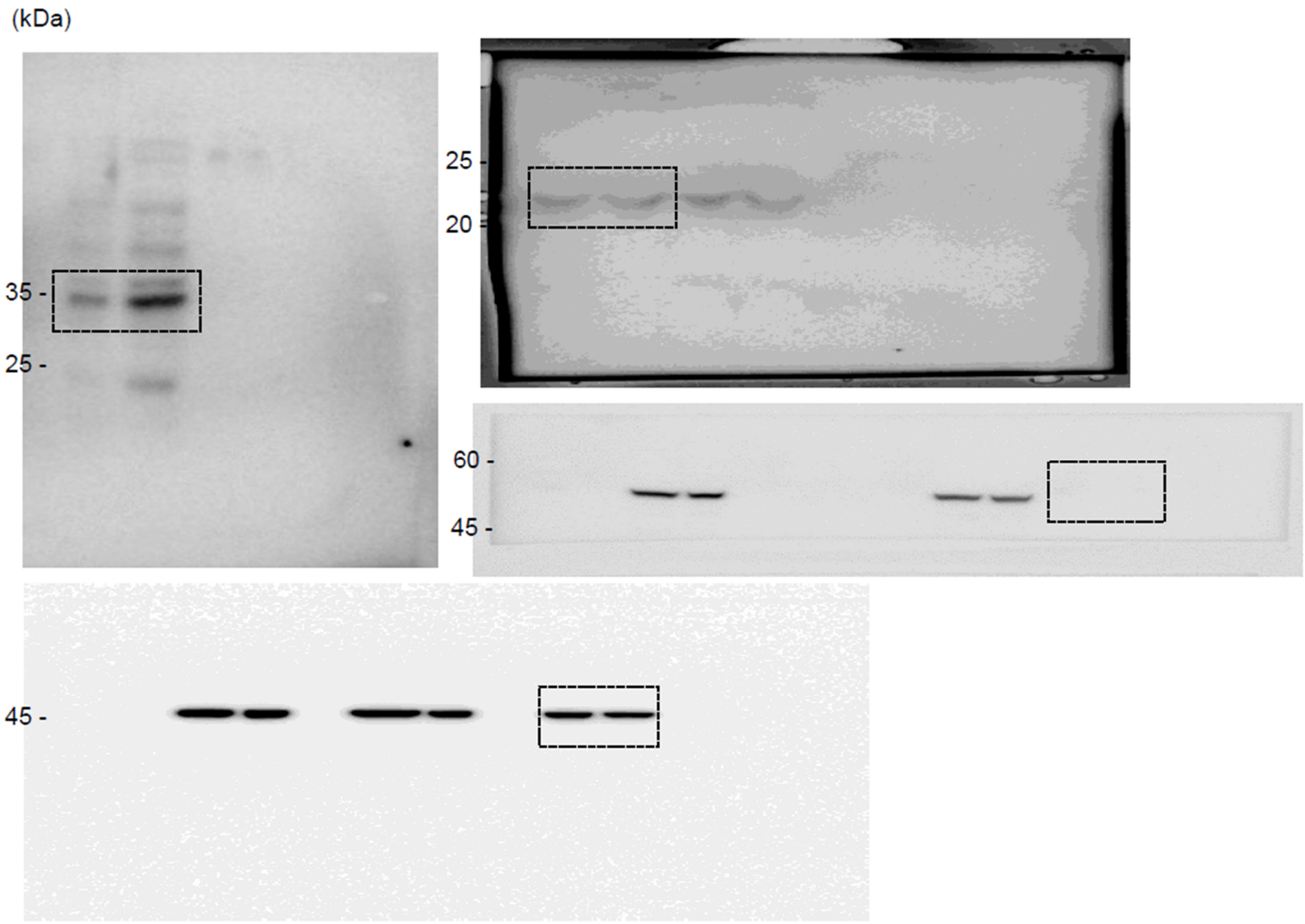


**Figure S3.** Uncropped western blots from Figure 3A. Uncropped western blot membranes, dotted lines indicate the cropped area used in Figure 3A western blot.

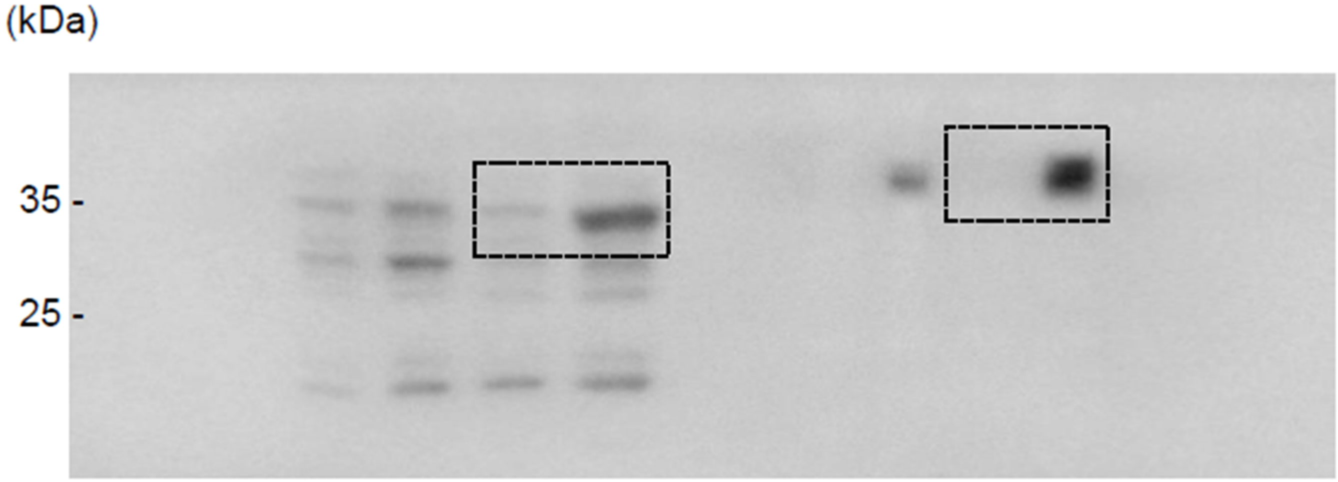


**Figure S4.** Uncropped western blots from Figure 4A. Uncropped western blot membranes, dotted lines indicate the cropped area used in Figure 4A western blot.

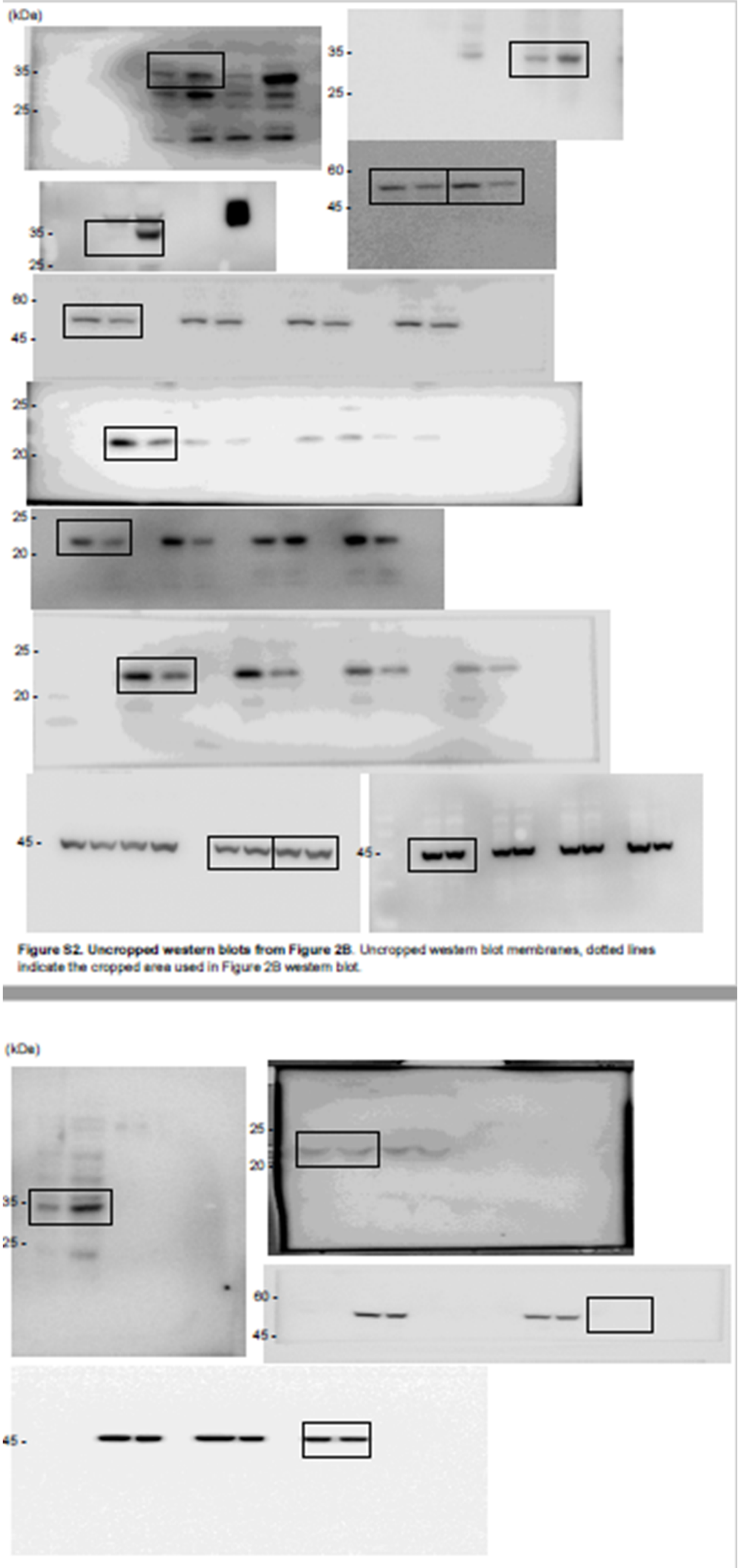


**Figure S5.** Uncropped western blots from Figure 4B. Uncropped western blot membranes, dotted lines indicate the cropped area used in Figure 4B western blot.
